# Knockdown of SIRT7 enhances the osteogenic differentiation of human bone marrow mesenchymal stem cells partly via activation of the Wnt/*β*-catenin signaling pathway

**DOI:** 10.1038/cddis.2017.429

**Published:** 2017-09-07

**Authors:** Erman E M Chen, Wei Zhang, Chenyi C Y Ye, Xiang Gao, Liangjun L J Jiang, Tengfei T F Zhao, Zhijun Z J Pan, Deting D T Xue

**Affiliations:** 1 Department of Orthopedics, Second Affiliated Hospital, School of Medicine, Zhejiang University, Hangzhou 310000, People’s Republic of China; 2 Orthopedics Research Institute of Zhejiang University, Hangzhou 310000, People’s Republic of China

## Abstract

Sirtuin 7 (SIRT7) is a NAD^+^-dependent deacetylase in the sirtuin family. In a previous study, human bone marrow mesenchymal stem cells (hBMSCs) with reduced SIRT7 activity were developed to evaluate the effect of SIRT7 on osteogenesis. SIRT7 knockdown significantly enhanced osteoblast-specific gene expression, alkaline phosphatase activity, and mineral deposition *in vitro*. Additionally, SIRT7 knockdown upregulated *β*-catenin. The enhanced osteogenesis due to SIRT7 knockdown was partially rescued by a Wnt/*β*-catenin inhibitor. Furthermore, SIRT7 knockdown hBMSCs combined with a chitosan scaffold significantly promoted bone formation in a rat tibial defect model, as determined by imaging and histological examinations. These findings suggest that SIRT7 has an essential role in osteogenic differentiation of hBMSCs, partly by activation of the Wnt/*β*-catenin signaling pathway.

Bone marrow stem cells (BMSCs) possess self-renewal capabilities and the potential to differentiate into a variety of cell types, including osteoblasts, chondrocytes, and adipocytes.^1^ BMSCs are considered promising seed cells for repair of bone defects. A better understanding of the osteogenesis of MSCs is necessary for clinical applications in bone regeneration.^2^ Thus discerning the genetic factors involved in MSCs osteogenesis is an important area of research.

SIRT7 is a member of the sirtuin family of histone deacetylases, the members of which have diverse roles in aging, DNA repair, cell cycle, metabolism, and disease biology.^3, 4, 5^ SIRT7 binds to the ribosomal RNA gene and is associated with reactivation of ribosomal DNA transcription following mitosis.^6^ Barber *et al.*^7^ revealed that SIRT7 is a NAD+-dependent H3K18Ac (acetylated lysine 18 of histone H3) deacetylase that stabilizes the transformed state of cancer cells. Moreover, several studies reported that SIRT7 is overexpressed in breast cancer tissues and in thyroid and colon tumors compared with normal tissue,^7, 8, 9^ and it showed a close relationship with inflammatory cardiomyopathy and apoptosis.^10^ In addition, sirtuin family genes and osteogenic differentiation are closely related. SIRT1 is a regulator of bone mass and a repressor of sclerostin.^11^ SIRT3 is required for osteogenesis via modulation of mitochondrial function and biogenesis via PGC-1*α*/SOD2 signaling.^12^ Sun *et al.*^13^ showed an essential role of SIRT6 in osteogenesis of rat MSCs partly by suppressing NF-*κ*B signaling. However, little is known about the role of SIRT7 in the osteogenesis of MSCs.

Wnt/*β*-catenin signaling is a crucial regulator of MSCs that has important roles in osteogenic differentiation.^14, 15^ The canonical Wnt signaling pathway is activated upon Wnt binding to frizzled receptors^16^ and LRP co-receptor at the cell membrane,^17, 18^ causing inhibition of glycogen synthase kinase-3*β* (GSK3*β*) and stabilizing cytoplasmic *β*-catenin.^15^ Via activation of Wnt, *β*-catenin accumulates in the cytosol and translocates to the nucleus where it promotes T-cell factor/lymphoid enhancing factor-1-mediated transcription,^19, 20^ thereby affecting target gene transcription. Moreover, several secreted molecules are antagonists of Wnt signaling, including secreted glycoproteins dickkopfs (Dkks) and secreted frizzled-related proteins.^17, 21^ Of these, Dkk1 was shown to inhibit the canonical Wnt signaling pathway in several different cell types.^22, 23, 24, 25^ Previous studies have shown that Wnt signaling is critical for bone formation and maintenance and that canonical signaling promotes stromal progenitor proliferation and osteogenesis.^26, 27, 28^ Additionally, the sirtuin family and Wnt signaling are closely related. SIRT1 promotes bone formation by preventing *β*-catenin sequestration by FoxO transcription factors.^29^ As a tumor suppressor, SIRT2 inhibits the Wnt signaling pathway by binding to *β*-catenin produced in response to radiation-induced stress.^30^ Wang *et al.*^31^ found that SIRT6 regulates hematopoietic stem cell homeostasis and self-renewal capacity via Wnt signaling.

In this study, we investigated the expression of SIRT7 in human BMSCs (hBMSCs) and its effect on the osteogenic differentiation of hBMSCs. We found that endogenous expression of SIRT7 decreased during osteogenic differentiation. We hypothesized that downregulation of SIRT7 promotes osteogenesis of hBMSCs via Wnt/*β*-catenin signaling. By assessing the expression levels of specific osteogenic markers and calcium deposition, we revealed that SIRT7 knockdown enhances osteogenic differentiation of hBMSCs partly via the Wnt/*β*-catenin signaling pathway *in vitro*. We also used a rat tibial defect model transplanted with SIRT7 knockdown hBMSCs, combined with the use of a chitosan scaffold, and found that SIRT7 knockdown promoted healing of bone defects *in vivo*.

## Results

### Endogenous SIRT7 expression

**Figure 1:**
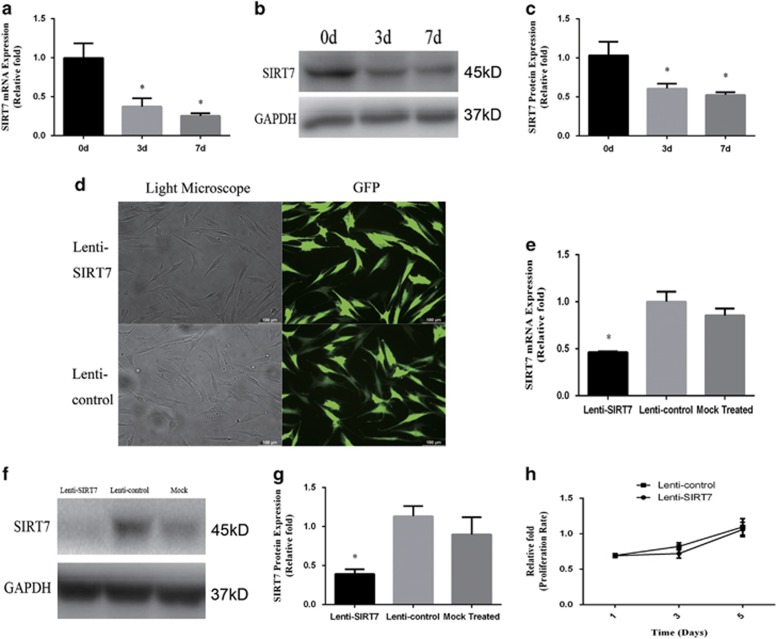
Endogenous SIRT7 expression and the construction of SIRT7-konckdown hBMSCs and lenti-control hBMSCs. (**a**–**c**) The endogenous expression of SIRT7 mRNA and protein were determined, respectively, by qPCR and western blotting analysis at days 0, 3, and 7 of osteogenic differentiation. (**d**) hBMSCs after lentiviral transfection and puromycin screening were observed under a normal microscope and a fluorescence microscope. (**e**–**g**) The mRNA and protein levels of SIRT7 were determined, respectively, by qPCR and western blotting analysis among the lenti-SIRT7, lenti-control group, and mock treated group. (**h**) The proliferation rate of hBMSCs was not significantly affected by SIRT7 knockdown. The mRNA and protein expression levels were normalized to glyceraldehyde 3-phosphate dehydrogenase (GAPDH). All the data were confirmed by three repeated tests. The data are expressed as means±S.D., *P<0.05 *versus* the lenti-control group

To determine the expression levels of SIRT7 associated with osteogenic differentiation of MSCs, we examined SIRT7 endogenous expression in hBMSCs at days 0, 3, and 7 during osteogenic differentiation. Compared with undifferentiated hBMSCs, both the mRNA (Figure 1a) and protein expression (Figures 1b and c) of SIRT7 was decreased significantly at days 3 and 7.

### SIRT7 knockdown in hBMSCs

To clarify the role of SIRT7 in osteogenic differentiation, a lentiviral vector system was used to efficiently knock down SIRT7 of third-generation hBMSCs. SIRT7 knockdown was quantified by evaluating the ratio of green fluorescent protein (GFP)-positive cells to the total cell number (Figure 1d). SIRT7 expression was quantified by real-time PCR and western blotting analysis 3 days after infection and screening. Compared with the lenti-control and mock-treated groups (without virus), SIRT7 was decreased in the lenti-SIRT7 group (Figures 1e and f). The mRNA and protein expression of SIRT7 knockdown hBMSCs at passage 9 retained a stable knockdown efficiency (see Supplementary Figure 1).

### SIRT7 knockdown did not affect hBMSC proliferation

To determine whether SIRT7 knockdown influences the proliferation of hBMSCs, we measured CCK8 levels in hBMSCs. The effects of SIRT7 knockdown on hBMSC proliferation at days 1, 3, and 5 after infection are shown in Figure 1h. No significant difference was detected in the cell proliferation rate between SIRT7 knockdown and lenti-control hBMSCs.

### SIRT7 knockdown increased the levels of osteo-specific genes and proteins

**Figure 2:**
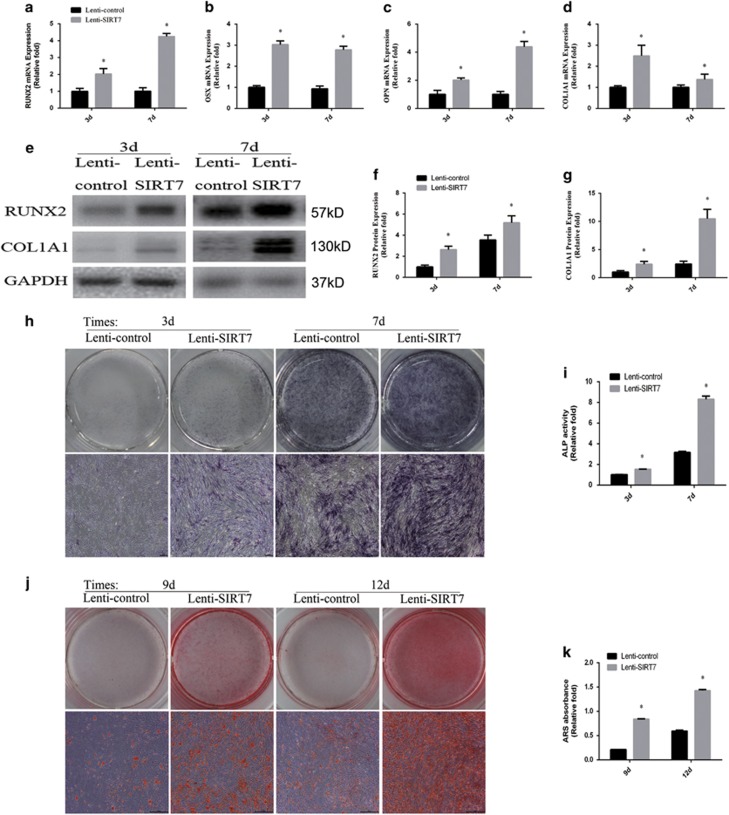
The knockdown of SIRT7 promoted osteogenic differentiation of hBMSCs. (**a**) The expression of RUNX2 mRNA was determined by qPCR at days 3 and 7 of osteogenic differentiation. (**b**) The expression of OSX mRNA. (**c**) The expression of OPN mRNA. (**d**) The expression of COL1A1 mRNA. (**e**–**g**) The expression of RUNX2 and COL1A1 proteins were determined by western blotting analysis after osteogenic differentiation for 3 and 7 days. (**h**) ALP in hMSCs was stained after the osteogenic differentiation for 3 and 7 days. (**i**) The ALP activity of hMSCs. (**j**) ARS after the osteogenic differentiation for 9 and 12 days. (**k**) Mineralization was quantified by the extraction of ARS-stained cells. All the data were confirmed by three repeated tests. Data were mean±S.D. *P<0.05 *versus* the lenti-control group. Scale bar=200 *μ*m

To evaluate the role of SIRT7 knockdown in osteogenic differentiation, the levels of osteo-specific genes and proteins, including runt-related transcription factor 2 (RUNX2), osterix (OSX), osteopontin (OPN), and collagen type I alpha 1 chain (COL1A1), were determined by quantitative real-time PCR (qPCR) and western blotting analyses. qPCR analysis revealed that RUNX2, OSX, OPN, and COL1A1 mRNA levels were significantly higher in SIRT7 knockdown hBMSCs than in the control group at days 3 and 7 (*P*<0.05, Figures 2a–d). Western blotting analysis revealed higher RUNX2 and COL1A1 protein expression in SIRT7 knockdown hBMSCs than in control cells (Figure 2e).

**Figure 3:**
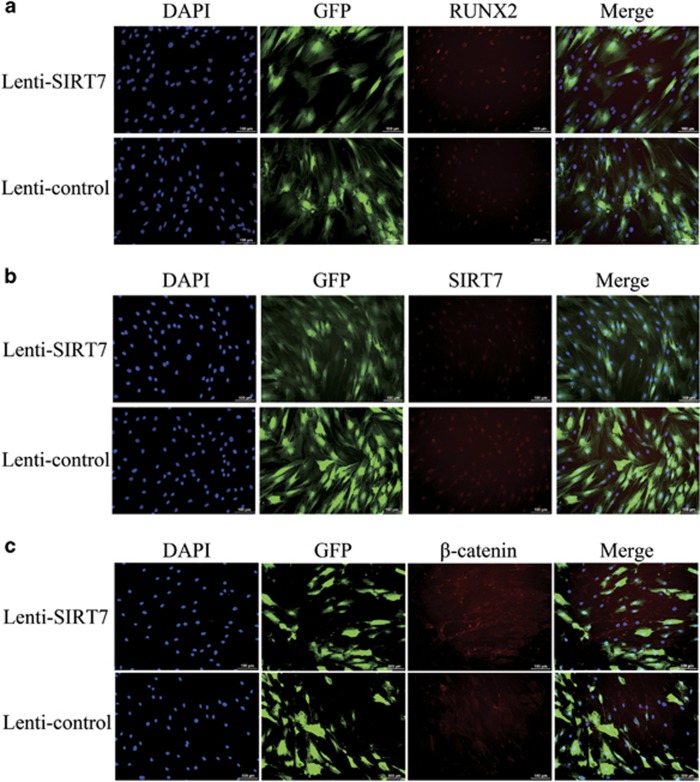
Immunofluorescence staining showed the protein levels of RUNX2, SIRT7, and *β*-catenin. (**a**) The level of RUNX2 protein (red) at day 3 of osteogenic differentiation. (**b**) The level of SIRT7 protein (red) at day 3 of osteogenic differentiation. (**c**) The level of *β*-catenin protein (red) at day 3 of osteogenic differentiation. The nuclei were counterstained with DAPI (4′,6-diamidino-2-phenylindole; blue). All the data were confirmed by three repeated tests. Scale bar=100 *μ*m

We also performed an immunofluorescence analysis to assess RUNX2 protein expression, and the results showed that RUNX2 expression levels were increased on day 3 in SIRT7 knockdown cells (Figure 3a). SIRT7 protein expression was very low on day 3 in SIRT7 knockdown cells, which indicates efficient knockdown during osteogenesis (Figure 3b).

### SIRT7 knockdown enhanced alkaline phosphatase (ALP) activity and calcium deposit formation

ALP activity is a marker of early-stage osteogenesis. We evaluated ALP activity at days 3 and 7 during osteogenic differentiation. Compared with the control group, higher ALP activity was observed in the SIRT7 knockdown group (*P*<0.05, Figure 2i). Similar results were observed by ALP staining (Figure 2h). Calcium deposits were also examined by Alizarin red staining (ARS), and the stained areas were quantified by measuring the absorbance at 560 nm. More calcium deposits were discovered in the SIRT7 knockdown than in the control group at days 9 and 12 (Figure 2j). The quantification analysis produced similar results (Figure 2k).

### SIRT7 knockdown activated the Wnt/β-catenin signaling pathway

**Figure 4:**
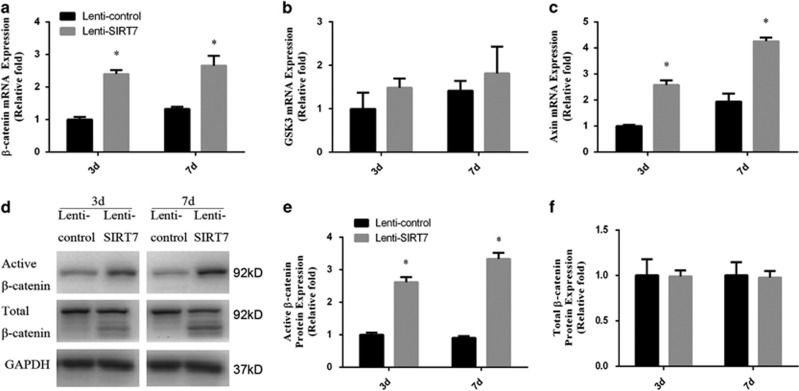
The knockdown of SIRT7 upregulated the Wnt/*β*-catenin signaling pathway during osteogenesis. (**a**) The expression of *β*-catenin mRNA was determined by qPCR at days 3 and 7 of osteogenic differentiation. (**b**) The expression of GSK3*β* mRNA. (**c**) The expression of Axin mRNA. (**d**–**f**) The expression of active *β*-catenin and total *β*-catenin proteins were determined by western blotting analysis at days 3 and 7 of osteogenic differentiation. All the data were confirmed by three repeated tests. Data were mean±S.D. *P<0.05 *versus* the lenti-control group

To confirm the above findings suggesting a role for Wnt/*β*-catenin signaling, the expression of *β*-catenin was determined by qPCR, western blotting analysis, and immunofluorescence analysis during osteogenesis at days 3 and 7. The expression of GSK3*β* and Axin was also determined by qPCR. The results of the qPCR and western blotting analyses revealed higher expression of *β*-catenin in the SIRT7 knockdown hBMSCs (Figures 4a and d). Compared with the control group, Axin level was upregulated in SIRT7 knockdown hBMSCs; however, GSK3*β* level did not change (Figures 4b and c). Moreover, immunofluorescence analysis showed higher levels of *β*-catenin accumulation in the cytoplasm in the SIRT7 knockdown group at day 3 (Figure 3c).

### Increased osteogenic differentiation of SIRT7 knockdown hBMSCs was partially rescued by a Wnt/β-catenin inhibitor

**Figure 5:**
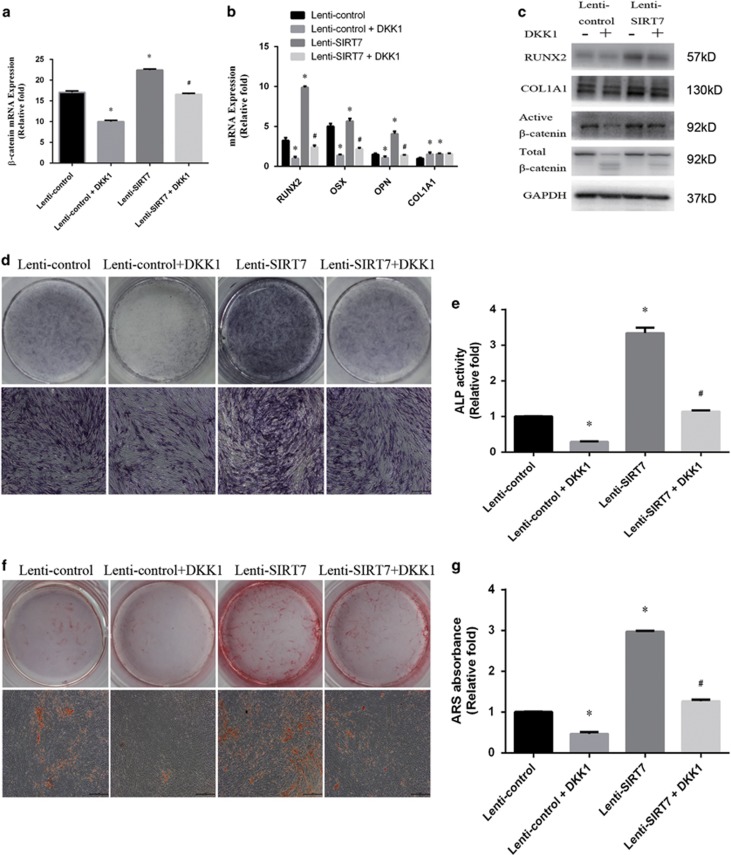
The increased osteogenesis caused by SIRT7 konckdown could be rescued partially by the addition of a Wnt/*β*-catenin signaling inhibitor (DKK1). (**a**) The expression of *β*-catenin, GSK3*β*, and Axin mRNA in the lenti-control, lenti-control+DKK1, lenti-HSPA1A, and lenti-HSPA1A+DKK1 groups were determined by qPCR at days 3 of osteogenesis. (**b**) The expression of RUNX2, OSX, OPN, and COL1A1 mRNA at days 3 of osteogenesis. (**c**) The expression of RUNX2, COL1A1, active *β*-catenin, and total *β*-catenin proteins were determined by western blotting analysis at day 3 of osteogenesis. (**d** and **e**) ALP staining and activity at day 7 of osteogenesis. (**f** and **g**) ARS and quantitation at day 9 of osteogenesis. All the data were confirmed by three repeated tests. Data were mean±S.D. *P<0.05 *versus* the lenti-control group. #*P*<0.05 *versus* the lenti-SIRT7 group. Scale bar=200 *μ*m

To confirm involvement of the Wnt/*β*-catenin signaling pathway, we examined the effect of Wnt/*β*-catenin inhibition on osteogenesis in the SIRT7 knockdown cells. After the addition of DKK1 for 3 days, the level of non-phosphorylated (active) *β*-catenin was significantly decreased compared with the level in SIRT7 knockdown hBMSCs without the inhibitor (Figures 5a and c). Moreover, inhibition of Wnt/*β*-catenin partially reversed the increase in osteogenesis of hBMSCs, as indicated by the expression of osteo-specific genes and proteins (Figures 5b and c). In addition, ALP activity and staining revealed higher ALP activity in SIRT7 knockdown hBMSCs than in the lenti-SIRT7+DKK1 group (Figures 5d and e). ARS staining to determine the amount of calcium deposits showed similar results (Figure 5f and g).

### A chitosan scaffold combined with the SIRT7 knockdown hBMSCs accelerated bone fracture healing in a rat tibial defect model

**Figure 6:**
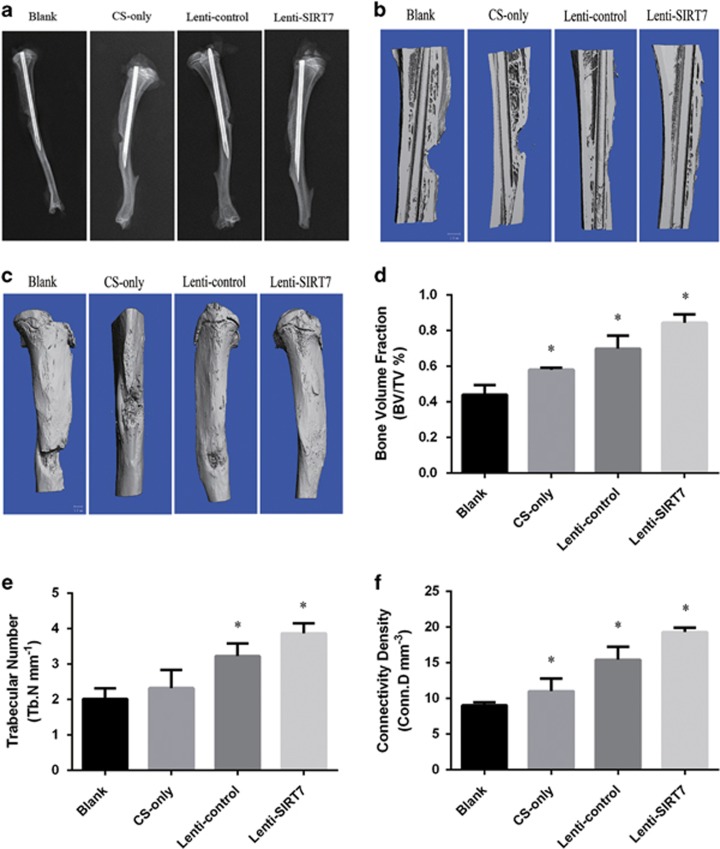
Radiographic and micro-CT analyses of the defect area at 6 weeks after surgery in each group. (**a**) Radiographic analysis. (**b** and **c**) Micro-CT images. (**d**–**f**) Micro-CT analyses of BV/TV, Tb.N, and Conn.D. All the data were confirmed by three repeated tests. Data were mean±S.D. *P<0.05 *versus* the blank group. Scale bar=1 mm

Radiographs taken at 6 weeks showed that the cortical defect was clearly present in the blank group. In the chitosan scaffold-only and lenti-control groups, this gap was obscured, and a greater amount of bridging callus formation was evident in the defect area compared with that in the blank group. In the SIRT7 knockdown hBMSC group, the gap had almost disappeared (Figure 6a). Micro-computed tomography (micro-CT) revealed significantly more new bone formation in the SIRT7 knockdown hBMSC group than in the chitosan scaffold-only and lenti-control groups; among the four groups, the largest defect appeared in the blank group (Figures 6b and c). Quantitatively, the fractures in the SIRT7 knockdown group displayed a significant increase in the bone volume fraction (BV/TV), trabecular number (Tb.N), and connectivity density (Conn.D) compared with the blank group (Figures 6d–f).

### Histological analysis of bone regeneration

**Figure 7:**
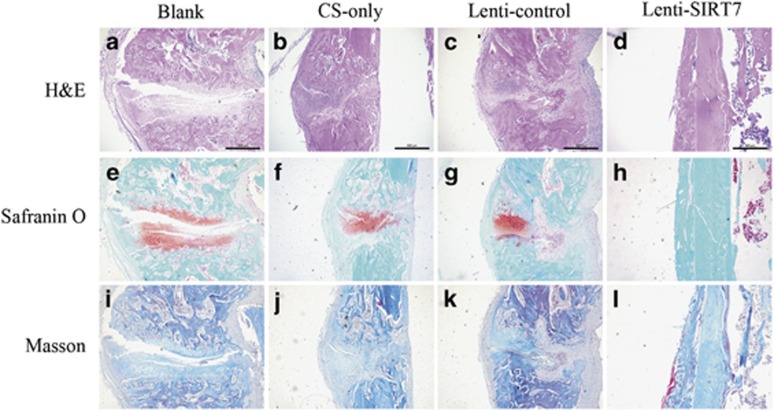
Histological evaluation of the defect area at 6 weeks after surgery in each group. (**a**–**d**) HE staining. Scale bar=500 *μ*m. (**e**–**h**) Safranin O and fast green staining. (**i**–**l**) Masson staining. All the data were confirmed by three repeated tests. Scale bar=500 *μ*m

Histological analyses are shown for each cell group in Figure 7. Sections were stained with hematoxylin and eosin, Safranin O and Fast green, and Masson stain. No bridging bone formation in the defect area was observed in the blank group (Figures 7a, e, and i). In the chitosan scaffold-only and lenti-control groups, a thick callus consisting of newly formed woven bone tissue was found in the defect area (Figures 7b, c, f, g, j, and k). In the SIRT7 knockdown hBMSC group, the defect area was almost sealed and remodeling of the callus was almost completed, indicating bony healing of the defect (Figures 7d, h, and l).

## Discussion

To our knowledge, this is the first study to explore the effects of SIRT7 on MSC osteogenic differentiation. We found that endogenous expression of SIRT7 was downregulated in hBMSCs during osteogenesis. Therefore, we used a SIRT7 knockdown strategy to promote osteogenic differentiation of MSCs. We found that SIRT7 knockdown accelerated osteogenesis of hBMSCs partly via the Wnt/*β*-catenin signaling pathway *in vitro*. Moreover, SIRT7 knockdown hBMSCs combined with a chitosan scaffold accelerated bone fracture healing in a rat bone defect model. These findings indicate that SIRT7 knockdown enhances osteogenesis of hBMSCs, at least partly via activation of the Wnt/*β*-catenin signaling pathway.

Yeast silent information regulator 2 (Sir2), a member of the sirtuin family, was originally identified in a screening for silencing factors.^32^ Imai *et al.*^33^ found that Sir2 functions as a NAD-dependent histone deacetylase involved in metabolism, aging, and genomic silencing in yeast. Surprisingly, studies of other model organisms (including *Caenorhabditis elegans* and *Drosophila*) also indicated Sir2 homologs as determinative factors of lifespan.^34^ Prokaryotic and eukaryotic sirtuins are homologous with yeast Sir2, and seven mammalian homologs have been identified so far.^35^ Among these, three mammalian sirtuins (SIRT1, SIRT6, and SIRT7) are located in the nucleus, with SIRT1 also found in the cytoplasm. SIRT7 is located in the nucleolus in human cells and associates with ribosomal DNA and interacts with RNA polymerase I.^35^ Ford *et al.*^36^ found that SIRT7 activity requires the amino-acid residues that bind to NAD within the conserved sirtuin core domain, indicating a role for NAD-dependent regulation. SIRT7-deficient mice experience a reduction in lifespan and progressive heart hypertrophy and inflammatory cardiomyopathy.^10^ Overexpression of SIRT7 exhibits oncogenic properties, such as promotion of colony formation, a more invasive phenotype, and cell growth both *in vitro* and *in vivo*.^37^ A recent report showed that SIRT7 acted as an oncogene in gastric cancer and hepatocellular carcinoma.^38, 39^ Recently, SIRT7, the least-investigated member of the sirtuin family, has been a focus of research of diseases, such as hypertrophic inflammatory cardiomyopathy, fatty liver disease, oncogenic transformation, tumor growth, and age-related hearing loss.

Immunofluorescence analysis showed that SIRT7 is located in the nucleus, consistent with the observation of Ford *et al.*^36^ The effect of SIRT7 in hBMSCs during osteogenesis was evaluated by qPCR and western blotting analyses, which showed endogenous SIRT7 expression to be significantly downregulated at days 3 and 7 after osteogenic differentiation. We constructed SIRT7 knockdown hBMSCs for additional experiments. ALP staining and calcium deposits are regarded as early and late markers of osteoblastic differentiation, respectively.^40^ We found that SIRT7 knockdown enhanced ALP activity and accelerated mineralization. RUNX2, a master transcription factor in osteogenic differentiation,^41^ was significantly increased in expression following knockdown of SIRT7, as determined by qPCR, western blotting, and immunofluorescence analysis. Other osteogenic markers, such as OSX, Col1, and OPN, showed similar patterns. Meanwhile, SIRT7 knockdown did not affect proliferation of hBMSCs. These results suggest that SIRT7 knockdown promotes osteogenesis of hBMSCs *in vitro*.

Wnt/*β*-catenin signaling is an essential signaling pathway required for bone formation.^42^ Under the influence of the Wnt ligand, frizzled and LRP-5/6 co-receptors recruit Disheveled to the plasma membrane together with Axin-GSK3*β*, inhibiting formation of the *β*-catenin complex and increasing *β*-catenin levels in the cytoplasm.^15, 43, 44^ This leads to nuclear translocation of *β*-catenin and activation of target genes.^45^ In our present study, we observed that SIRT7 knockdown increased the expression of *β*-catenin during osteogenesis, and immunofluorescence analysis confirmed these observations. Furthermore, the increased osteogenesis of hBMSCs by SIRT7 knockdown was partially rescued by an inhibitor of Wnt/*β*-catenin (DKK1). Knockdown of *β*-catenin by RNA interference using small interfering RNAs produced similar results. These findings indicate that knockdown of SIRT7 regulates osteogenic differentiation of hBMSCs via activation of the Wnt/*β*-catenin signaling pathway.

A previous report demonstrated that porous chitosan-alginate scaffolds are osteoconductive, and experimental treatments showed improved defect closure in a calvarial defect model.^46, 47^ Polyampholytic chitosan fibers promoted proliferation and osteogenic differentiation of MSCs, as well as osseous tissue regeneration, in a rabbit model.^48^ In our study, the use of a porous chitosan scaffold with hBMSCs promoted bone healing in a rat tibial defect model. Better bone formation was observed when SIRT7 knockdown hBMSCs were present in the scaffold.

Many studies have demonstrated a relationship between expression of sirtuins and stem cell osteogenesis. However, this is the first study to demonstrate the impact of SIRT7 on osteogenic differentiation of MSCs. Unfortunately, we determined the effect of only SIRT7 knockdown, and not SIRT7 overexpression, on osteogenesis. Moreover, the mechanisms of activation of the Wnt/*β*-catenin signaling pathway by SIRT7 knockdown are not fully clarified, especially with regard to the nuclear translocation of *β*-catenin. Other signaling pathways need to be examined for potential involvement in the osteogenesis of hMSCs by SIRT7 knockdown in future studies.

## Conclusion

According to our results, SIRT7 knockdown enhanced osteogenic differentiation of hBMSCs, partly via activation of the Wnt/*β*-catenin signaling pathway. SIRT7 knockdown in hBMSCs combined with a chitosan scaffold enhanced bone defect repairs and may provide a new stem cell-based strategy for bone regeneration.

## Materials and methods

### Cell culture and reagents

hBMSCs, purchased from Cyagen Biosciences (Guangzhou, China), can differentiate into osteoblasts, adipocytes, and chondrocytes under specific inductive conditions. Adherent hBMSCs were cultured in culture flasks in hMSC growth medium (Cyagen Biosciences, Inc., Guangzhou, China) in an incubator at 37 °C with 5% CO_2_ and were passaged after reaching 80% confluence. Cells from passages 3–9 were used in subsequent experiments. Recombinant DKK1 was purchased from PeproTech (Rocky Hill, NJ, USA). Based on a previous study, we used a DKK1 of 0.5 *μ*g/ml.^49^

### Lentiviral packaging and cell infection

Lentiviral knockdown SIRT7 (lenti-SIRT7) particles and lentiviral GFP particles, used as the control group (lenti-control), were prepared by GenePharma Co., Ltd (Shanghai, China). For infections, 40–60% confluent hBMSCs were incubated with lentiviral particles and 2.5 *μ*g/ml polybrene in growth medium at a multiplicity of infection of 50. After 12 h, >95% of the cells were still viable, and the culture medium was then changed. Three days later, all transfected cells were passaged for use in subsequent experiments. The expression of SIRT7 was determined by qPCR and western blotting analyses.

### Cell viability assay

To assess the effect of SIRT7 knockdown on the proliferation of hBMSCs, cells were seeded into a 96-well plate (5000/well) and allowed to adhere for 24 h. After 24 h, the medium was removed, and the cells were treated with 10% Cell Counting Kit-8 (CCK-8, Dojindo, Kumamoto, Japan) in 100 *μ*l low-sugar Dulbecco’s modified Eagle’s medium (L-DMEM) without fetal bovine serum (FBS) for 3 h at 37 °C. Absorbance at 450 nm, which is directly proportional to cell proliferation, was measured using a microplate reader (ELX808; BioTek, Winooski, VT, USA).

### Osteogenic differentiation protocol

hMSCs were cultured in growth medium (L-DMEM; 10% FBS (1495527; Gibco, Waltham, MA, USA) and 100 IU/ml penicillin/streptomycin) in 6- or 12-well cell culture plates at a density of 3 × 10^4^/cm^2^ and incubated for 48 h at 37 °C under 5% CO_2_. The cells were subsequently cultured in osteogenic induction medium (L-DMEM with 10% FBS, 100 IU/ml penicillin/streptomycin, 100 nM dexamethasone, 0.2 mM ascorbic acid, and 10 mM *β*-glycerophosphate). The cells were maintained by the addition of fresh osteogenic induction medium every 2–3 days.

### ALP staining and ALP activity assay

Cells were cultured in osteogenic induction medium in 12-well plates for 3 or 7 days. For ALP staining, cells were fixed with 4% paraformaldehyde for 15 min. Cells were then washed twice with PBS and stained using the BCIP/NBT Alkaline Phosphatase Color Development Kit (Beyotime, Shanghai, China). For measurement of ALP activity, cells were lysed with lysis buffer consisting of 20 mM Tris–HCl (pH 7.5), 150 mM NaCl, and 1% Triton X-100. ALP activity was determined using the ALP Activity Assay (Beyotime) according to the manufacturer’s instructions. Briefly, the conversion of colorless p-nitrophenyl phosphate to colored p-nitrophenol was measured after 3 and 7 days of culture in osteogenic medium at 405/650 nm.

### Alizarin red staining

After induction of osteogenic differentiation, mineral deposition was assessed by ARS (Cyagen Biosciences). Cells were fixed in 4% paraformaldehyde for 15 min at room temperature and subsequently washed with distilled water. The cells were incubated with a 0.5% solution of alizarin red for 20–30 min at room temperature, followed by rinsing with distilled water. The stain was desorbed by incubating with 10% cetylpyridinium chloride (Sigma, Shanghai, China) for 1 h. The solution was collected, and 200 *μ*l were plated on 96-well plates, which were read at 560 nm using a microplate reader (ELX808; BioTek). The readings were normalized to the total protein concentration.

### RNA isolation and qPCR

Total cellular RNA was isolated using RNAiso reagent (Takara, Dalian, China) and quantified by measuring the absorbance at 260 nm (NanoDrop 2000; Thermo Fisher Scientific, Waltham, MA, USA). First-strand cDNA was synthesized using PrimeScript RT Master Mix (Takara) according to the manufacturer’s instructions. Total RNA (⩽1000 ng) was reverse-transcribed into cDNA in a reaction volume of 20 *μ*l using the Double-Strand cDNA Synthesis Kit (Takara). One microliter of cDNA was used as the template for qPCR. All gene transcripts were quantified by qPCR using the Power SYBR Green PCR Master Mix (Takara) on the ABI StepOnePlus System (Applied Biosystems, Warrington, UK). The mRNAs of the target genes and the housekeeping gene (GAPDH) were quantified in separate tubes. All primers were synthesized by Sangon Biotech (Shanghai, China). The primer sequences used are shown in Table 1. The cycle conditions were as follows: 95 °C for 30 s, followed by 40 cycles at 95 °C for 5 s and 60 °C for 30 s. The relative target gene expression levels were calculated using the 2^−ΔΔCt^ method.

### Western blotting analysis

Cells were lysed in RIPA buffer supplemented with a proteasome inhibitor (Beyotime). Equal amounts of proteins were separated by 10% sodium dodecyl sulfate polyacrylamide gel electrophoresis and then transferred to a polyvinylidene fluoride membrane (Millipore, Shanghai, China). After blocking in 5% non-fat milk for 2 h, the membranes were incubated overnight at 4 °C with antibodies specific to GAPDH (1 : 1500; Cell Signaling Technology, Shanghai, China), SIRT7 (1 *μ*g/ml; Abcam, Shanghai, China), RUNX2 (1 : 1600; Cell Signaling Technology), COL1A1 (1 : 1000; Abcam), non-phosphorylated (active) *β*-catenin (1 : 1000; Cell Signaling Technology), or total *β*-catenin (1 : 1000; Cell Signaling Technology). After washing in TBST four times (5 min each), the membranes were incubated with horseradish peroxidase-conjugated secondary antibodies (anti-mouse or anti-rabbit; Beyotime) for 1 h at room temperature. After washing five times with TBST, we detected proteins using enhanced chemiluminescence blotting reagents according to the manufacturer’s instructions. The immunoreactive bands were detected using an enhanced chemiluminescent detection reagent (Millipore). Signal intensity was measured using the Bio-Rad XRS chemiluminescence detection system (Bio-Rad, Hercules, CA, USA).

### Immunofluorescence analysis

Cells were cultured in induction medium in a 12-well plate and evaluated for RUNX2, SIRT7, and *β*-catenin using a fluorescence microscope (EU5888; Leica, Wetzlar, Germany) as follows. Cells were fixed in 4% paraformaldehyde for 15 min at room temperature, permeabilized, and blocked for 30 min in 0.05% Triton X-100 and 2% bovine serum albumin. Fixed cells were washed and incubated overnight with anti-RUNX2 (1 : 1600; Cell Signaling Technology), SIRT7 (10 *μ*g/ml; Abcam), or non-phosphorylated (active) *β*-catenin (1 : 1600; Cell Signaling Technology). Cells were incubated with a fluorescence-conjugated secondary antibody (Beyotime) for 120 min, and nuclei were stained with 4′,6-diamidino-2-phenylindole (KeyGen Biotech, Nanjing, China) for 5 min. Samples were observed under a fluorescence microscope (Leica).

### Scaffold sterilization and hMSC seeding and culture

Chitosan scaffolds, which were gifts from Professor Hu, were sterilized by incubation in 70% ethanol for 30 min followed by UV exposure for 30 min on each side. The chitosan scaffolds (1 × 1 cm^2^) were placed in 24-well plates. Once placed into sterile tissue culture plates, scaffolds were incubated for 4 h in growth medium to remove any residual alcohol and to wet the scaffolds. Cells were seeded at a density of 50 000/scaffold in 50 *μ*l medium and incubated at 37 °C for 3 h to ensure attachment; the medium was then brought to 600 *μ*l/well. Medium was exchanged after 24 h and changed every day.

### *In vivo* evaluation in animals

All Sprague Dawley (SD) rats were supplied by the Academy of Medical Sciences of Zhejiang Province. All animal experiments were performed in accordance with the Animal Care and Use Committee guidelines of Zhejiang Province. All experimental procedures were approved by the Institutional Animal Care and Use Committee at Zhejiang University.

All surgical procedures were performed by two experienced traumatic orthopedic surgeons (ZJP and DTX). Tibial defects were generated in 8-week-old male SD rats (weighing approximately 200 g). The rats were anesthetized intraperitoneally with 0.3% pentobarbital sodium (Sigma) at 30 mg/kg body weight. After anesthesia, an incision was made below the knee. An intramedullary needle (1.2-mm-diameter stainless steel syringe needle) was inserted inside the medullary canal of the tibia for fixation. The tibial defect model was established as reported previously.^50, 51^ A 1.5-mm-diameter tibial defect was made in all SD rats approximately 5 mm from the proximal tibial growth plate by a hollow drill. The same leg was used for each group. The 24 defects in 24 rats were randomized into four groups. In the blank group (*n*=6), nothing was grafted onto the fracture site; in the negative-control group (*n*=6), the defect areas were filled with chitosan scaffold only; in the lenti-control group (*n*=6), the scaffolds seeded with lenti-control hBMSCs were implanted into the tibial defects; and in the lenti-SIRT7 group (*n*=6), the scaffolds with lenti-SIRT7 hBMSCs were implanted into the defects.

### Radiographic analysis and micro-CT evaluation

Animals were killed 6 weeks after surgery, and the samples were collected. Radiographs were taken using a dual-track molybdenum/rhodium+ Mo target mammography machine (22 KV, 250 mAS; GE, Fairfield, CT, USA) to evaluate callus formation at the defect site. All samples were scanned for bone formation using a micro-CT 100 imaging system (Scanco Medical, Brüttisellen, Switzerland) with the following scan parameters: 70 kVp X-ray energy, 1024 reconstruction matrix, 0.0148 mm slice thickness, and 300 ms integration time. BV/TV, Tb.N, and Conn.D were calculated by three-dimensional standard microstructural analysis.^52^

### Histological evaluation

The samples were fixed in 4% paraformaldehyde for 72 h at room temperature and then decalcified using 10% EDTA (Sigma), changing the solution once a week for >8 weeks, before embedding in paraffin. Serial sections of 3-*μ*m thickness were cut and mounted on polylysine-coated slides. Hematoxylin and eosin, Safranin O and Fast green, and Masson staining were performed separately on consecutive tissue sections. For hematoxylin and eosin staining, tissue sections after deparaffinization were rehydrated and stained with hematoxylin for 30 s, rinsed in water for 1 min, eosin for 10–30 s, and dehydrated with alcohol. For Safranin O and Fast green staining, tissue sections after deparaffinization were rehydrated and stained with hematoxylin for 30 s, Fast green for 15 min, rinsed in PBS for 15 min, Safranin O for 5 min, and dehydrated with alcohol. For Masson staining, tissue sections after deparaffinization were rehydrated and stained with hematoxylin for 30 s, Biebrich scarlet-acid fuchsin for 5 min, rinsed in PBS for 15 min, phosphomolybdic acid for 5 min, rinsed in PBS for 15 min, Aniline blue for 5 min, and dehydrated with alcohol. Images were obtained using a microscope.

### Statistical analysis

Statistical analysis was performed using the SPSS 17.0 software (IBM, Armonk, NY, USA). All experiments were performed in at least triplicate, and the data are presented as means±S.D.

Statistical significance was determined using a two-tailed Student’s *t*-test when comparing two groups and one-way ANOVA followed by Bonferroni’s *post hoc* test when comparing more than two groups. A *P*-value⩽0.05 was considered to represent a statistically significant difference.

## Publisher’s Note

Springer Nature remains neutral with regard to jurisdictional claims in published maps and institutional affiliations.

## Figures and Tables

**Table 1:** Sequences of primers for quantitative real-time PCR

**Gene name**	**Forward primer sequence (5′→3′)**	**Reverse primer sequence (5′→3′)**
SIRT7	CAGGGAGTACGTGCGGGTGT	TCGGTCGCCGCTTCCCAGTT
RUNX2	ACTTCCTGTGCTCGGTGCT	GACGGTTATGGTCAAGGTGAA
OSX	CCTGCGACTGCCCTAATT	GCGAAGCCTTGCCATACA
OPN	ATGATGGCCGAGGTGATAGT	ACCATTCAACTCCTCGCTTT
COL1A1	GAGAGCATGACCGATGGATT	CCTTCTTGAGGTTGCCAGTC
*β*-Catenin	TTAAGCCTCTCGGTCTGTGG	GCCGCTTTTCTGTCTGGTTC
GSK3	GACTAAGGTCTTCCGACCCC	TTAGCATCTGACGCTGCTGT
Axin	TTATGCTTTGCACTACGTCCCTCCA	CGCAACATGGTCAACCCTCAGAC
GAPDH	GAAAGCCTGCCGGTGACTAA	TGGAATTTGCCATGGGTGGA
