# Supplementary figures and images for: Knockdown of SIRT7 enhances the osteogenic differentiation of human bone marrow mesenchymal stem cells partly via activation of the Wnt/β-catenin signaling pathway
Source: Cell Death Dis. 2017 Sep 7;8(9):e3042–. doi: 10.1038/cddis.2017.429 (PMC5636975; doi:10.1038/cddis.2017.429)

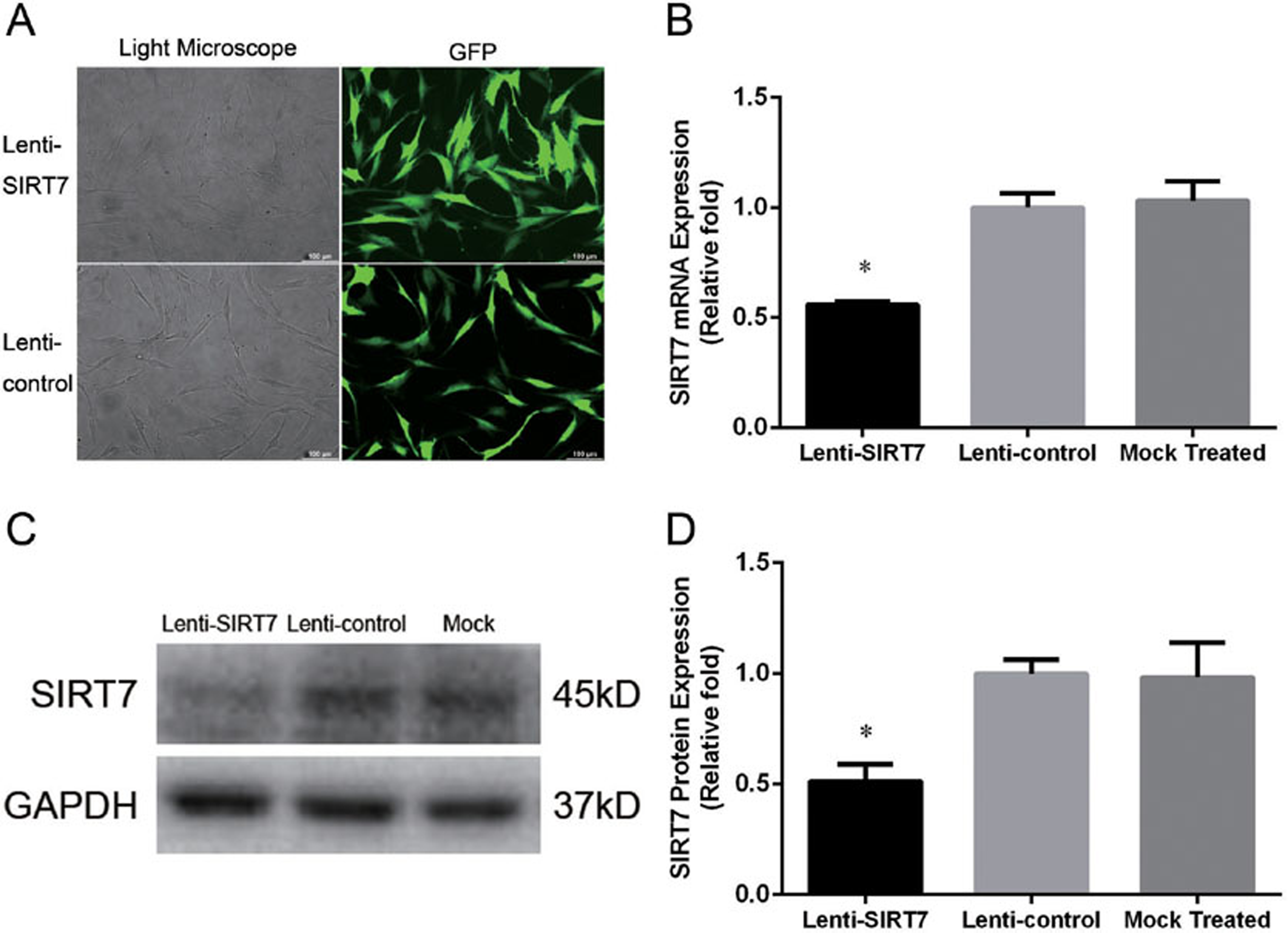

Supplement: Supplementary Figure 1 [file cddis2017429x1.tif]
