# Supplementary material for: Knockdown of SIRT7 enhances the osteogenic differentiation of human bone marrow mesenchymal stem cells partly via activation of the Wnt/β-catenin signaling pathway
Source: Cell Death Dis. 2017 Sep 7;8(9):e3042–. doi: 10.1038/cddis.2017.429 (PMC5636975; doi:10.1038/cddis.2017.429)
Supplement: Supplementary Figure 1 Legend [file cddis2017429x2.docx]

Figure 1. The SIRT7 expression of SIRT7-konckdown hBMSCs at passage 9. (A) hBMSCs of lentiviral transfection at passage 9 were observed under a normal microscope and a fluorescence microscope. (B-D) The mRNA and protein levels of SIRT7 were determined respectively by qPCR and Western blot analysis among the lenti-SIRT7, lenti-control group, and mock treated group. All the data were confirmed by three repeated tests. The data are expressed as means ± standard deviation, * p < 0.05 vs. the lenti-control group.
